# Supplementary material for: The Role of Kidney Function in Predicting COVID-19 Severity and Clinical Outcomes: A Retrospective Analysis
Source: Infect Dis Rep. 2025 Jul 7;17(4):79. doi: 10.3390/idr17040079 (PMC12286045; doi:10.3390/idr17040079)
Supplement: Supplementary file 1 [file idr-17-00079-s001.zip › idr-3692372-supplementary.pdf]

Supplementary file

Table S1. Shapiro-Wilk normality test for demographic, clinical, and laboratory parameters.

	Total (n=359)		eGFR < 60 (n=161)		eGFR > 60 (n=198)	
	Shapiro-Wilk		Shapiro-Wilk		Shapiro-Wilk	
	W	p-value	W	p-value	W	p-value
Age	0.991	0.021	0.982	0.030	0.988	0.082
SBP	0.993	0.171	0.994	0.800	0.984	0.037
DBP	0.988	0.012	0.982	0.074	0.979	0.011
MAP	0.989	0.016	0.986	0.166	0.980	0.014
Heart Rate	0.977	0.000	0.962	0.000	0.970	0.000
Shock Index	0.875	0.000	0.824	0.000	0.976	0.006
Temperature	0.982	0.000	0.964	0.002	0.988	0.176
Respiratory Rate	0.948	0.000	0.936	0.000	0.954	0.000
SpO_2	0.735	0.000	0.701	0.000	0.798	0.000
ROX	0.130	0.000	0.975	0.018	0.124	0.000
pH	0.278	0.000	0.901	0.000	0.171	0.000
pCO_2	0.917	0.000	0.917	0.000	0.897	0.000
pO_2	0.814	0.000	0.834	0.000	0.796	0.000
Bicarbonate	0.979	0.000	0.989	0.389	0.949	0.000
Base Excess	0.865	0.000	0.964	0.001	0.683	0.000
Lactate	0.585	0.000	0.517	0.000	0.794	0.000
Arterial blood glucose	0.758	0.000	0.709	0.000	0.817	0.000
Hemoglobin	0.961	0.000	0.981	0.029	0.930	0.000
Hematocrit	0.966	0.000	0.985	0.089	0.939	0.000
Leukocytes	0.834	0.000	0.796	0.000	0.881	0.000
Band neutrophils	0.479	0.000	0.508	0.000	0.474	0.000
Neutrophils	0.851	0.000	0.815	0.000	0.893	0.000
Eosinophils	0.374	0.000	0.531	0.000	0.335	0.000
Basophils	0.773	0.000	0.789	0.000	0.241	0.000
Lymphocytes	0.890	0.000	0.894	0.000	0.880	0.000
Atypical lymphocytes	0.264	0.000	0.247	0.000	0.278	0.000
Monocytes	0.782	0.000	0.717	0.000	0.880	0.000
Neutrophil-to-lymphocyte ratio	0.041	0.000	0.737	0.000	0.520	0.000
Platelets	0.932	0.000	0.942	0.000	0.903	0.000
Platelets-to-lymphocytes ratio	0.736	0.000	0.803	0.000	0.652	0.000
C-reactive protein	0.869	0.000	0.839	0.000	0.894	0.000
Urea	0.763	0.000	0.868	0.000	0.888	0.000
Creatinine	0.491	0.000	0.592	0.000	0.991	0.241
Urea-to-creatinine	0.914	0.000	0.950	0.000	0.862	0.000

ratio						
Sodium	0.977	0.000	0.970	0.001	0.981	0.008
Potassium	0.962	0.000	0.985	0.100	0.984	0.027
ALT	0.228	0.000	0.212	0.000	0.573	0.000
D-Dimer	0.573	0.000	0.590	0.000	0.593	0.000

SBP = systolic blood pressure; DBP = diastolic blood pressure; MAP = mean arterial blood pressure; SpO_2 = peripheral capillary oxygen saturation; ROX = Respiratory rate-oxygenation index; ALT = alanine aminotransferase. Linear regression analyses were performed for the total cohort, as well as for subgroups with eGFR < 60 and eGFR > 60 mL/min/m^2. p-values > 0.05 indicate that the data do not significantly deviate from a normal distribution.

Table S2. Linear regression of GFR on demographic data.

Variables	Total (n=359)			eGFR < 60 (n=161)			eGFR ≥ 60 (n=198)		
	Adjusted R^2	F-test	*p*-value	Adjusted R^2	F-test	*p*-value	Adjusted R^2	F-test	*p*-value
Sex	0.006	3.023	0.083	-0.006	0.125	0.725	-0.005	0.030	0.862
Age	0.042	16.830	<0.001	0.047	8.805	0.003	0.249	66.160	<0.001
SAH	0.028	11.330	<0.001	0.009	2.404	0.123	0.065	14.670	<0.001
DM	-0.002	0.443	0.500	0.001	1.179	0.279	0.009	2.731	0.100
Cardiovascular disease	0.014	6.034	0.015	-0.006	0.087	0.768	0.032	7.402	0.007
Cerebrovascular disease	0.000	1.103	0.294	-0.002	0.721	0.397	-0.004	0.229	0.633
CKD	0.152	65.190	0.001	0.136	26.240	<0.001	0.004	1.811	0.180
Immunosuppression	0.037	14.660	<0.001	0.004	1.637	0.203	0.021	5.138	0.025
COPD	-0.002	0.140	0.709	0.005	1.851	0.176	0.009	2.708	0.101
Asthma	0.003	1.926	0.166	-0.004	0.315	0.575	-0.005	0.002	0.966
Other respiratory illness	-0.003	0.031	0.861	-0.004	0.395	0.530	-0.005	0.026	0.873
Neoplasia	0.000	1.705	0.301	-0.474	0.251	0.617	0.003	1.529	0.218
Solid organ transplantation	0.075	30.130	<0.001	0.005	1.739	0.189	0.008	2.606	0.108
Obesity	-0.002	0.108	0.743	0.001	1.239	0.267	0.029	6.932	0.009
Smoking	0.008	3.721	0.055	-0.005	0.215	0.644	0.003	0.163	0.204
Previous hospitalization	-0.003	0.001	0.978	0.008	2.270	0.134	0.001	1.219	0.271

SAH = systemic arterial hypertension; DM = diabetes mellitus; CKD = chronic kidney disease; COPD = chronic obstructive pulmonary disease.

Table S3. Linear regression analysis of vital signs according to eGFR values.

	Total (n=359)			eGFR < 60 (n=161)			eGFR ≥ 60 (n=198)		
	Adjusted R^2	F-test	*p*-value	Adjusted R^2	F-test	*p*-value	Adjusted R^2	F-test	*p*-value
SBP (mmHg)	0.011	4.596	0.033	0.018	3.451	0.065	-0.001	0.787	0.376
DBP (mmHg)	0.041	14.150	<0.001	0.002	1.317	0.253	0.009	2.619	0.107
MAP (mmHg)	0.032	11.130	<0.001	0.010	2.323	0.130	0.006	2.043	0.155
Heart rate (bpm)	0.036	12.640	<0.001	-0.006	0.188	0.665	0.085	17.140	<0.001
Shock Index	-0.003	0.005	0.945	0.018	3.481	0.064	0.030	6.128	0.014
Temperature (ºC)	-0.002	0.332	0.565	-0.002	0.732	0.394	-0.005	0.270	0.604
Respiratory rate (bpm)	-0.003	0.006	0.937	-0.007	0.087	0.769	-0.005	0.169	0.682
SpO_2 (%)	-0.001	0.547	0.460	-0.007	0.000	0.986	-0.005	0.022	0.883
ROX Index	-0.002	0.308	0.579	-0.005	0.351	0.555	-0.005	0.135	0.713

SBP = systolic blood pressure; DBP = diastolic blood pressure; MAP = mean arterial pressure; SpO_2 = peripheral capillary oxygen saturation; ROX = Respiratory rate-oxygenation index. All variables are reported as the median and interquartile range [Q1; Q3].

Table S4. Linear regression analysis of laboratory parameters according to eGFR values.

	Total (n=369)			eGFR < 60 (n=161)			eGFR ≥ 60 (n=198)		
	Adjusted R^2	F-test	*p*-value	Adjusted R^2	F-test	*p*-value	Adjusted R^2	F-test	*p*-value
pH	0.009	3.768	0.053	0.198	34.070	<0.001	0.000	1.027	0.312
pCO_2 (mmHg)	0.014	5.197	0.023	-0.001	0.914	0.341	0.010	2.621	0.108
pO_2 (mmHg)	-0.002	0.381	0.537	0.021	3.832	0.052	0.001	1.186	0.278
Bicarbonate (mEq/L)	0.225	85.560	<0.001	0.149	24.400	<0.001	0.034	6.450	0.012
Base excess	0.273	109.600	<0.001	0.228	40.480	<0.001	0.029	5.629	0.019
Lactate (mg/dL)	-0.003	0.207	0.649	-0.007	0.024	0.878	-0.005	0.209	0.648
Arterial blood glucose (mg/dL)	-0.002	0.567	0.452	-0.002	0.764	0.384	0.006	1.799	0.182
Hemoglobin (g/dL)	0.100	40.500	<0.001	0.118	22.310	<0.001	0.000	0.915	0.340
Hematocrit (%)	0.078	30.890	<0.001	0.107	20.040	<0.001	0.001	1.273	0.261
Leukocytes (/μL)	-0.003	0.077	0.781	-0.004	0.349	0.556	0.012	3.341	0.065
Band neutrophils (/μL)	0.008	3.834	0.051	-0.006	0.022	0.881	-0.005	0.000	0.990
Neutrophils (/μL)	-0.002	0.294	0.588	-0.001	0.835	0.362	0.014	3.776	0.053
Eosinophils (/μL)	-0.001	0.482	0.488	0.027	5.436	0.021	0.012	3.301	0.071
Basophils (/μL)	0.013	5.631	0.018	-0.006	0.100	0.753	-0.005	0.009	0.893
Lymphocytes (/μL)	0.020	8.220	0.004	0.009	2.464	0.118	-0.002	0.656	0.419
Atypical Lymphocytes (/μL)	-0.002	0.309	0.579	-0.006	0.009	0.924	0.001	1.187	0.277
Monocytes (/μL)	-0.003	0.055	0.815	0.000	0.955	0.330	0.005	1.894	0.170
Neutrophil-to-lymphocyte ratio	0.015	6.247	0.013	-0.005	0.202	0.654	-0.005	0.054	0.817
Platelets (/μL)	0.005	2.853	0.092	0.002	1.381	0.242	0.006	2.170	0.142
Platelet-to-lymphocyte ratio	0.011	4.946	0.027	-0.003	0.461	0.498	-0.003	0.385	0.536
C-Reactive protein (mg/L)	-0.002	0.503	0.479	0.011	2.479	0.118	-0.007	0.000	0.992
Urea (mg/dL)	0.561	456.900	<0.001	0.444	128.900	<0.001	0.245	64.450	<0.001
Creatinine (mg/dL)	0.387	226.700	<0.001	0.450	131.700	<0.001	0.487	187.700	<0.001
Urea-to-creatinine ratio	0.006	3.029	0.083	0.156	30.610	<0.001	0.006	2.214	0.138

Sodium (mEq/L)	0.006	3.319	0.069	-0.002	0.695	0.406	-0.005	0.055	0.815
Potassium (mEq/L)	0.195	84.830	<0.001	0.058	10.420	0.002	0.059	12.980	<0.001
ALT (U/L)	-0.004	0.036	0.849	-0.006	0.361	0.549	-0.007	0.067	0.796
D-Dimer (μg/mL)	0.047	13.360	<0.001	0.023	3.688	0.057	0.015	3.148	0.078

pO_2 = partial pressure of oxygen; pCO_2 = partial pressure of carbon dioxide; ALT = alanine aminotransferase.
